# The Role of Cell Metabolism in Skin Wound Healing

**DOI:** 10.34133/research.1000

**Published:** 2025-11-19

**Authors:** Wenshuai Liu, Haiyue Jiang, Dongliang Yang, Yannan Xie

**Affiliations:** ^1^ Plastic Surgery Hospital, Chinese Academy of Medical Sciences and Peking Union Medical College, Beijing 100144, China.; ^2^ Key Laboratory of External Tissue and Organ Regeneration, Chinese Academy of Medical Sciences and Peking Union Medical College, Beijing 100144, China.; ^3^ Key Laboratory of Flexible Electronics (KLOFE) and Institute of Advanced Materials (IAM), School of Physical and Mathematical Sciences, Nanjing Tech University (NanjingTech), Nanjing 211816, China.; ^4^ State Key Laboratory of Organic Electronics and Information Displays and Institute of Advanced Materials (IAM), Jiangsu Key Laboratory for Biosensors, Jiangsu National Synergetic Innovation Center for Advanced Materials (SICAM), Nanjing University of Posts and Telecommunications, Nanjing 210023, China.

## Abstract

Since the skin is the largest organ of the human body, skin injuries may result in substantial social and economic burdens. Skin wound management remains a substantial challenge due to the complex nature of the healing process involving inflammation, tissue proliferation, and remodeling. Coordinated interactions between cells, extracellular matrices, and cytokines drive regeneration, while cell metabolism—an essential pillar of physiological activity—regulates key biological processes like proliferation, angiogenesis, immune responses, and tissue regeneration. Modulating metabolic pathways provides a promising alternative to conventional therapies. However, understanding of the role of cell metabolism in wound healing remains to be fully elucidated. Further exploration of their cross talk will not only establish a robust theoretical foundation for innovative treatments but also open avenues for promoting scar-minimizing regenerative healing and bringing new prospects for clinical practice.

## Introduction

Skin injury incidence has risen annually due to trauma, disease prevalence, and aging, making wound repair a global healthcare priority [1,2]. Wound healing is a sophisticated, multistage process that relies on the synchronized actions of keratinocytes, fibroblasts, vascular endothelial cells, and macrophages, alongside signaling pathways and extracellular components, determining regeneration or fibrosis outcomes. Cellular metabolism has emerged as a fundamental regulator, supplying energy, biosynthetic precursors, and signaling molecules essential for repair. Wound repair induces local and systemic metabolic changes, demanding continuous replenishment of various cellular contents, organelles, phospholipid membranes, adenosine triphosphate (ATP), and other essential substances [3–6]. Despite its importance, metabolic rules during healing are not fully clarified, necessitating focused research on metabolic regulation’s impact on wound healing.

Nutrients like glucose and protein play a critical role in supporting effective repair. Glucose metabolism adapts dynamically across healing stages to meet energy and intermediate demands [7], while protein metabolism complements glucose metabolism, supporting the synthesis of metabolic enzymes, inflammatory mediators, growth factors, and collagen. Protein malnutrition impairs collagen synthesis and maturation, emphasizing the need for adequate intake to maintain tissue integrity [8–10].

**Fig. 1.**
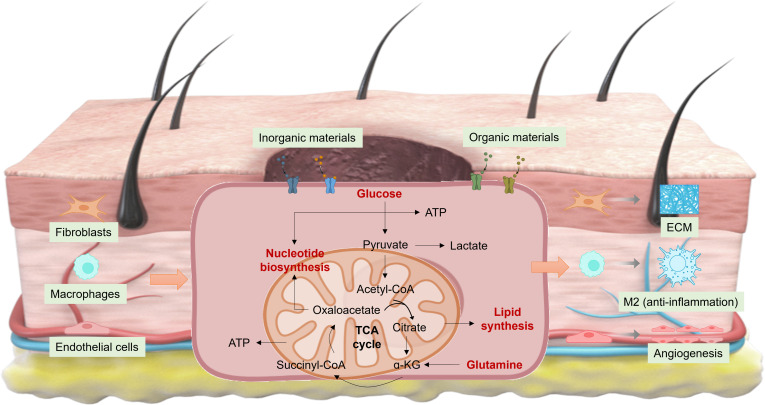
Cell metabolism reprogramming in skin wound healing. ATP, adenosine triphosphate; CoA, coenzyme A; TCA, tricarboxylic acid; α-KG, α-ketoglutarate; ECM, extracellular matrix.

In late acute and chronic wounds, glycolysis, the tricarboxylic acid cycle (Fig. 1), glutamine catabolism, and fatty acid oxidation are upregulated [11]. Glycolysis and glutaminolysis appear to exert particularly significant influences on healing efficiency, which are rapidly activated in the hypoxic/nutrient-limited injured microenvironment to generate ATP quickly, providing immediate energy for “emergency repair processes” in the early stage of injury, such as inflammatory cell recruitment and cell migration. In contrast, oxidative phosphorylation and fatty acid β-oxidation depend on sufficient oxygen and an intact mitochondrial structure with a slow activation rate. This positions glycolysis and glutamine metabolism as promising targets for metabolic intervention. In this perspective, we highlight key metabolic adaptations during skin repair, which may open new avenues for therapeutic innovation.

## Biomaterials for Metabolic Regulation

Innovative biomaterials targeting cellular metabolism and mitochondrial function offer great potential for repairing large-area wounds from severe diseases or traumatic injuries. Inorganic materials like nano-sized gadolinium oxide, carbon nanotubes, and hydroxyapatite promote bone metabolism and regeneration by enhancing mitochondrial function and oxidative metabolism [12]. Moreover, metal ions , such as iron, copper, and magnesium, play a fundamental role in metabolic regulation, serving as enzyme cofactors, catalysts in metabolic pathways, and electron acceptors in numerous cellular metabolic reactions. Iron ions are essential trace elements in the human body, playing crucial roles in DNA synthesis, vitamin A metabolism, and neurotransmitter synthesis [13]. Copper ions are constituents of proteins such as oxidase and superoxide dismutase, which are involved in a wide range of cellular metabolic pathways, including energy metabolism, oxidative stress response, vascular endothelial growth factor secretion, and iron metabolism [14]. Cu^2+^-loaded hydrogels modulate glycolysis, inhibit M1 macrophage polarization, and protect mitochondrial function; magnesium participates in protein synthesis and ATP production [15]. Similarly, magnesium ions participate in protein synthesis, DNA synthesis, and ATP production [16]. In summary, metal ions are indispensable for the regulation of metabolism.

Moreover, endogenous metabolites, such as succinate, fructose 1,6-bisphosphate, and α-ketoglutarate, enable in situ metabolic regulation. Professor Lu innovatively designed a high-energy fructose hydrogel [17]. This hydrogel can regulate glucose metabolism in situ, efficiently converting cellular energy, enhancing ATP synthesis, and accelerating the healing process. Modulating key metabolic enzymes, such as hexokinase, phosphofructokinase, isocitrate dehydrogenase, and succinate dehydrogenase, can enhance mitochondrial energy metabolism. Additionally, strategies to improve mitochondrial energy metabolism include activating the AMPK and nuclear factor E2-related factor 2 signaling pathways, stimulating silent information regulators to maintain mitochondrial functional homeostasis, and upregulating the expression of peroxisome proliferator-activated receptor γ coactivator 1-α to promote mitochondrial biogenesis. Bioactive factors such as melatonin and basic fibroblast growth factor protect mitochondria and promote repair in intervertebral disks and spinal cord injuries [18]. These biomaterial-based metabolic interventions highlight a shift toward precision modulation of wound microenvironments, holding great potential for overcoming conventional wound care limitations.

## Summary and Outlook

Metabolic reprogramming plays a pivotal role in wound repair via immune modulation and metabolite communication, with glucose, lipid, and amino acid metabolism increasingly showing promise for accelerating wound healing and reducing scarring. However, existing research in this area is not yet comprehensive enough to fully address the clinical challenges posed by various refractory wounds. Metabolic regulation orchestrates immune response, cell proliferation, angiogenesis, and tissue remodeling through precise regulation of energy metabolism and material metabolism; metabolic dysregulation induces chronic wounds. Systematic analysis of metabolic rewiring will inform targeted interventions like metabolic modulators and smart biomaterials. Cross-disciplinary insights from immunology and cancer metabolism offer valuable paradigms.

Metabolic imbalance in the wound microenvironment is a key driver of impaired healing, characterized primarily by insufficient energy supply and dysregulation of metabolite homeostasis [19,20]. Current biomaterials lack dynamic adaptation to phase-specific metabolic demands and cannot meet complex clinical needs. Future innovations should integrate multidisciplinary technical approaches, including synthetic biology (engineered bacteria), material informatics (artificial intelligence-optimized release curves), and microfluidic technology (personalized dressings) for spatiotemporal metabolic modulation. Despite unresolved questions, unraveling metabolic interactions between genes, molecules, and dressing technologies will drive therapeutic advancements. A metabolic-centric approach will alleviate nonhealing wound burdens, support regeneration in challenging clinical scenarios, and improve patient outcomes, opening new frontiers in wound repair research and translation.
